# Carbon-Nano Fibers Yield Improvement with Iodinated Electrospun PVA/Silver Nanoparticle as Precursor via One-Step Synthesis at Low Temperature

**DOI:** 10.3390/polym14030446

**Published:** 2022-01-22

**Authors:** Saharman Gea, Boy Attaurrazaq, Suhut Alexander Situmorang, Averroes Fazlur Rahman Piliang, Sunit Hendrana, Stergios Goutianos

**Affiliations:** 1 Cellulosic and Functional Materials Research Centre, Universitas Sumatera Utara, Jl. Bioteknologi No. 1, Medan 20155, Indonesia; boyattaurrazaq37@gmail.com (B.A.); suhutalexander09@gmail.com (S.A.S.); averroesfp@gmail.com (A.F.R.P.); 2 Department of Chemistry, Faculty of Mathematics and Natural Sciences, Universitas Sumatera Utara, Jl. Bioteknologi No. 1, Medan 20155, Indonesia; 3 Department of Physics, Faculty of Mathematics and Natural Sciences, Universitas Sumatera Utara, Jl. Bioteknologi No. 1, Medan 20155, Indonesia; 4 Research Centre for Chemistry, Indonesian Institute of Sciences (LIPI), Kawasan Puspitek Gedung 452, Serpong, Tangerang Selatan, Banten 15310, Indonesia; hendrana2012@gmail.com; 5 Department of Manufacturing and Civil Engineering, Norwegian University of Science and Technology, NO-2802 Gjøvik, Norway; stergios.goutianos@ntnu.no

**Keywords:** carbon nanofibers, electrospinning, iodination, PVA/AgNO_3_

## Abstract

High temperature is required in carbon fiber synthesis in the carbonization step. However, direct high-temperature heating without the presence of additive materials would affect the yield and structure of carbon fibers produced. Thus, this study aims to synthesize carbon fibers from poly-vinyl alcohol (PVA), as the precursor and reducing agent, using silver nanoparticles (SNP) from silver nitrate (AgNO_3_) as additives. The pre-treatment of PVA was performed in three steps, i.e., mixing PVA/AgNO_3_, electrospinning, and iodination. The interaction of PVA and AgNO_3_ was assessed by FTIR, and SEM was used to characterize the electro-spun fibers prior and after iodination; Raman spectrophotometer was carried out to confirm the yield of carbon fibers. There was reduction in oxygen groups (3000–3800 cm^−1^) and emergence of –C=O (1100 cm^−1^) and –C=C– (1627 cm^−1^) functional groups, indicating formation of carbon layers. Based on the DT/GA results, the silver nanoparticles reduce the need of high temperature with optimum carbonization at 350 °C and lead to the formation of more regular graphene layers. Graphene layers with a size distribution of 0.438 nm and well-organized structures were successfully formed, and the Raman shifting showed higher intensities of G and G’ bands in the presence of Ag. Based on DT/GA results, the yield of carbon fibers with iodinated PVA fibers and SNP as additive had higher rates around 800 µg/min, reaching 33% at 500 °C. Thus, it is demonstrated that iodinated PVA/AgNO_3_ samples can significantly improve carbon fiber yield at low temperatures.

## 1. Introduction

The introduction of carbon nanofibers (CNF) as a reinforcement in composite materials in engineering applications (e.g., aerospace and automotive industries), supercapacitors, high-temperature fillers and catalysts [1,2,3] are attractive due to their high mechanical properties. The demand for CNF as reinforcing material is expected to increase, whereas the methods that have been extensively used to synthesize CNF like chemical vapor deposition (CVD) are considered to be complicated and relatively costly. Within CVD, the growth that is initiated from gaseous hydrocarbon compounds (methane, ethane, and carbon monoxide) as precursors requires high temperatures around 700–1500 K [1]. Therefore, many efforts have been attempted towards alternative methods to produce CNF with affordable precursors, as well as high yield (the amount of CNF obtained) and relatively low cost. The use of polymer and electrospinning respectively as a precursor and as a method have been extensively investigated.

Polymers with long hydrocarbon chains are commonly used as precursors in carbon fiber synthesis. Several polymers, including poly(acrylonitrile), PAN, [4], poly(furfuryl alcohol) [5], poly(vinylidene fluoride), PVDF, [6], poly(vinyl alcohol) [7], poly(amic acid), PAA, [8], poly(p-xylene tetrahydrothiophenium chloride),PXTC, [9], poly(vinylidene chloride),PVDC, [6], and phenolic resin [10], resulted in over 50% carbon yield when synthesized with acrylonitrile in cyanide acid (HCN) [11]. With high carbon content, up to 54.5%, for polyvinyl alcohol (PVA), this polymer is considered appropriate as a precursor, and, moreover, it has high solubility in water, it is non-toxic, and acts as a reduction agent [12,13,14]. Furthermore, these characteristics make the synthesis of carbon fibers more numerous, and decrease the processing cost. In addition, the presence of –OH groups within the PVA are easily separated from the polymer chain; however, the final degradation occurred above 400 °C [15]. Hence, the thermal carbonization above 400 °C of PVA may result in lower yield of CNF, and therefore an alternative pre-preparation method is needed to overcome this issue.

Several attempts to find alternative pre-preparation methods have been proposed, particularly based on functional components and the addition of metals via electrospinning and coating techniques [16,17,18]. As the formation of carbon fibers generally employ three steps, i.e., stabilization, carbonization, and sometimes graphitization to improve the electrical and mechanical properties [19], a series of pre-treatment steps are introduced to maintain stabilization that leads to an increase of CNF yield. The addition of metal ions has been reported to positively affect the thermal decomposition and carbonization of PVA [20]. The metals during electrospinning accelerate the graphitization process, as well as improve the electrical properties [20]. An earlier study reported a 48% carbon fiber yield increase with the presence of Ni in PVA; nevertheless, the carbonization still started at a high temperature of 1200 °C [14]. Although the -OH within the PVA may act as the reduction agent, not all metals can be reduced into nanoparticles, one of which is silver. Thus, the above explanation suggests the selection of certain metals that could be reduced into nanoparticles to increase the yield of CNF (affecting thermal decomposition). 

In the present work, AgNO_3_ is introduced as the source of silver nanoparticle (SNP) for the thermal decomposition of PVA precursor. Pre-treatment technique of iodination within PVA and electrospinning are proposed to create fibrous structure. To the best of our knowledge, this direct use of SNP as the metal catalyst to improve the yield is the first to be used.

## 2. Materials and Methods

### 2.1. Materials

The chemical reagents used were polyvinyl alcohol (PVA) atactic full hydrolyzed (MW = 60,000), silver nitrate, iodine and distilled water in analytically pure condition. All the chemical reagents and samples were supplied from Sigma-Aldrich Corporation.

### 2.2. Methods

#### 2.2.1. Preparation of 13% PVA and 13% PVA/0.2% AgNO_3_ Solutions

The preparation of the Polyvinyl alcohol (PVA) solution was a crucial procedure because the pre-treatment affects the electrospinning results. Thus, some trials were performed to obtain a homogenous solution, and some trials such as dissolution in distilled water for 4 h and ultrasonication for 2 h were carried out. As the addition of AgNO_3_ crystal had no effects on the dissolution process, the PVA/AgNO_3_ mixture was prepared by using the same procedure. PVA polymer is considered as a medium viscous polymer, high polymeric bonds, and this could affect the dissolution process in water [17]. PVA polarity behavior changes with increasing temperature due to dipole orientation. Thus, to obtain a homogeneous solution, longer time with constant stirring at temperature of 80–100 °C and ultrasonication treatment were performed [21,22,23].

The preparation of PVA solution followed previous studies [24,25]. In brief, 13 g PVA were dissolved in 100 mL distilled water and constantly stirred for 4 h at 90 °C, followed by ultrasonication for two hours. The solution was characterized for its viscosity and conductivity. The PVA/AgNO_3_ solution was made by preparing 10 mL of 13% PVA solution and 0.2 wt% of silver nitrate (AgNO_3_). They were mixed by constant stirring for 24 h at room temperature [26]. Finally, the viscosity and the conductivity of the mixture were analyzed.

#### 2.2.2. Fabrication of Electro-Spun the 13% PVA and 13% PVA/0.2% AgNO_3_ Fibers

Nanofibers were fabricated by firstly preparing 2 mL of 13 w/v% PVA solution inside a 10-mL syringe. Then, it was electro-spun at 17.6 kV with 0.9 mL/h discharge rate. The collector in the electrospinning was at a 10 cm distance and its rotational speed was 110 rpm [21]. The obtained electro-spun sheet samples were left at room temperature for 24 h. Finally, the samples were stored inside a silica-gel-containing desiccator to prevent any damages from the external environment. The same procedure was applied for the fabrication of nanofibers of 13%PVA/0.2% AgNO_3_ with a slight modification. 2 mL of samples were prepared for exactly the same of 17.6 kV, 10 cm of distances, 110 rpm rotational speed (23) with 0.6 mL/h discharge rate. The same collector, syringe type and storing procedure were used and applied.

#### 2.2.3. Iodination Treatment in 13% PVA and 13% PVA/0.2% AgNO_3_

The iodination procedure was carried out following previous studies [27,28]. In brief, electro-spun 13% PVA and 13% PVA/0.2%AgNO_3_ sheets were iodized by adding iodine crystals into a protected vessel for 24 h at 80 °C. 

#### 2.2.4. Carbon Fiber Synthesis from Iodinated PVA and PVA/AgNO_3_

Carbon fiber synthesis was performed following the method of Ju et al. (2017) [29] via two a two-step calcination process. It has been reported that the softening point of PVA fibers is at 215–224 °C. Thus, to obtain a stable process, pre-heat treatment should be employed before the carbonization of the PVA fibers due to the fusion of the fibers at high temperatures. First, 0.5 g of iodinated PVA or PVA/AgNO_3_ fiber were calcinated for 1 h at 180 °C under atmospheric conditions. Then, the second calcination step was carried out at 500 °C for 6 h with a 5 °C minute^−1^ heating rate under nitrogen condition. During the second calcination step, the applied nitrogen flow rate was with 200 mL/min. 

### 2.3. Characterization

The successful preparation of polymeric 13% PVA and 13%/0.2% AgNO_3_ solutions for electrospinning was evaluated by measuring the electrical properties via a conductometer (Milawukee/Mi 180 Bench Meter), and the viscosity via a HAAKE^TM^ Viscotester^TM^ 550 Rotational Viscometer. The morphological characteristics of both nanofibers samples before and after iodination were characterised via a Zeiss Scanning Electron Microscope (SEM), whereas the calculation of the fiber’s diameter was performed via the ImageJ software. The determination of thermal degradation of the electrospun nanofibers, and the calculation the CNF yield was performed via a TGA instrument (TGA SDT Q600 V20). The functional groups of both electrospun nanofibers and CNF were characterized by using Fourier Transform-Infra Red (FT-IR) Shimadzu IR prestige-21. The determination of graphene layers and size of CNF was performed via Transmission Electron Microscope (TEM) JEOL JEM 1400 with particle size distribution analysis via ImageJ software, whereas the spectrophotometer UV-Vis (Perkin Elemr, Lambda 35) was used to determine the presence of SNP. The Raman spectrophotometer (iHR320 HORIBA) was performed to confirm the carbon structures.

## 3. Results

**Figure 1:**
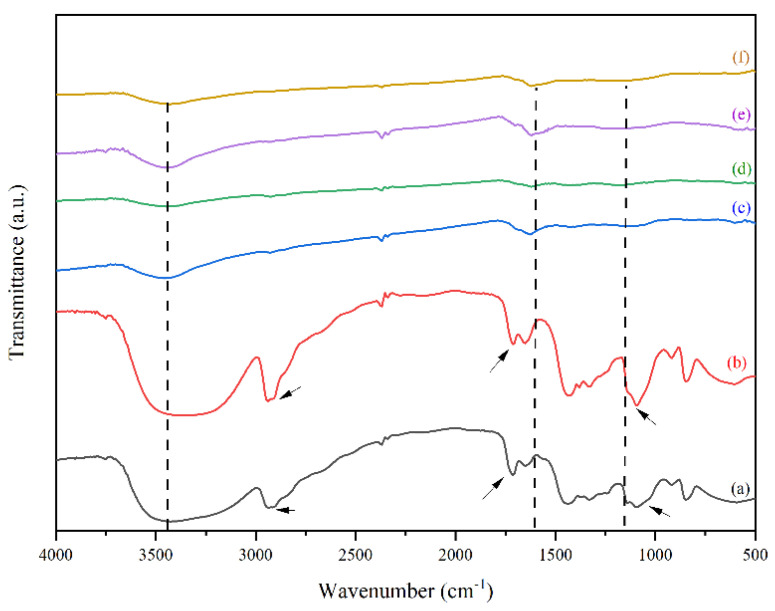
FTIR spectra of (**a**) neat PVA, (**b**) PVA/AgNO3 nanofiber, (**c**) Iodinated PVA/AgNO_3_ nanofiber, (**d**) Iodinated PVA nanofiber, (**e**) CNF, and (**f**) CNF/Ag.

Both physical and chemical interactions occurred when negative charges from hydroxyl groups in PVA interacted with the positive charges in Ag. The hydroxyl groups in PVA and the molecular groups from the solvent (water), as well as nitrate ion from AgNO_3_ were reported to produce hydrogen bonds [30] (additional file 1: Figure S1 in Supporting Data). The occurrence of these interactions was confirmed by FTIR spectra presented in Figure 1.

Based on Figure 1a,b, no noticeable differences occurred with the presence of AgNO_3_ in the PVA solution. It is assumed that the peak would normally exist in multiple band structures [31], thus; TEM measurements were carried out to detect the presence of silver. However, different transmittance of peaks could be observed in 1000–1600 cm^−1^ and 2750–3000 cm^−1^, which were stretching C–H and aromatic C–H respectively (indicated by the arrows in Figure 1). Similarly, the addition of AgNO_3_ to PVA solution had effects too on the –C=O and –C=C–, indicated by the peaks between 1600 cm^−1^ to 1700 cm^−1^. These showed redox reaction, where the oxidation state was indicated by the appearance of C = C and C = O functional groups as a result of hydroxy group oxidation in PVA. Meanwhile, the occurrence of oxidation caused other species to be reduced, in this case the Ag^+^ species were reduced to Ag^0^ which was shown by the changes in polymer color from transparent to brown color. These phenomena occurred due to the oxidation and dehydration of polyols in PVA molecules [27,32].

Figure 1c,d show the peaks of both iodinated PVA and PVA/AgNO_3_ have the same characteristics, such as the weakening of OH stretch band intensity at the peak around 3200–3700 cm^−1^, as well as stronger and wider intensity of C=C band at around 1600 cm^−1^. The reduction of OH stretch from Figure 1b,c confirms the reduction of oxygen groups that is caused by the iodination pre-treatment. Iodination that took place at 80 °C was considered as the result of polyol dehydrogenation in PVA and PVA/AgNO_3_ to become polyene [13,22,23].

Based on Figure 1d,e, the CNF spectra have similar characteristics to those in iodinated nanofiber samples (Figure 1b,c), suggesting that the iodination pre-treatment has a slight effect on the fiber functional groups. However, there were slight changes of intensity and more specific widening in the both CNF spectra (Figure 1d,e), particularly at 1627 cm^−1^ wavenumber, which was a C=C aromatic bond with hexagonal sp^2^ carbon formed [16]. The diameter size distribution of the electro-spun nanofiber and silver nanoparticles were observed clearly, which are indicated by the interconnection of enlarged fibres (Supplementary Data 1: Figure S2 in Supporting Data).

**Figure 2:**
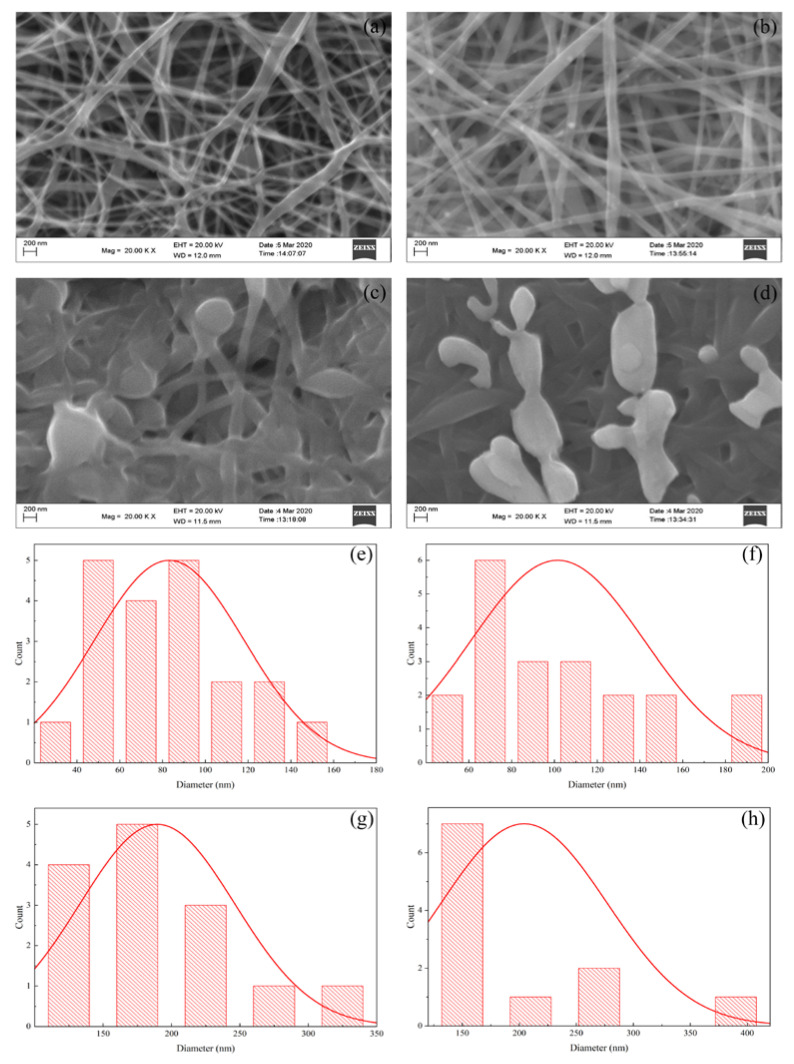
SEM morphological images of (**a**) PVA nanofiber; (**b**) PVA/AgNO_3_ Nanofiber; (**c**) iodinated PVA nanofiber; (**d**) iodinated PVA/AgNO_3_ nanofiber, and the diameter-size distributions of (**e**) PVA nanofiber; (**f**) PVA/AgNO_3_; (**g**) iodinated PVA nanofiber; and (**h**) iodinated PVA/AgNO_3_ nanofiber.

Figure 2 shows SEM images, where the deposition of Ag and iodinated effects on electro-spun nanofibers can be seen.

The analysis, using the Image-J software, showed that the PVA polymer produced nanofibers with uneven diameter sizes in between 20 and 160 nm (average 84.13 nm) as shown in Figure 2a. Meanwhile, the diameters of the PVA/AgNO_3_ fibers (Figure 2b) were significantly more uniform, as the addition of metals has increased the conductivity and the viscosity. The addition of AgNO_3_ resulted in an increase of the conductivity (Table 1) due to the presence of ionic constituents, such as Ag^+^ and NO_3_^−^, as the SEM images have shown the successful formation of nanofibers (both with and without iodination pre-treatment). The PVA/AgNO_3_ nanofibers before the iodination pre-treatment had a diameter between 40–200 nm (average 151.54 nm) which could be caused by the distribution of nanoparticles reduced by the polyol within the PVA. The increase in diameter and uniformity could be explained by the high viscosity and silver accumulation within the nanofibers that may deaccelerate the rate of electrospinning, as well as increase the conductivity (Figure 2b) [25]. Moreover, the addition of AgNO_3_ increased the Coulomb interaction which affects high viscosity solutions. As a result, the amount of PVA/AgNO_3_ that are allowed to pass on the edge of syringe could be controlled. The amount of AgNO_3_ at 0.2% of weight fraction appeared to be the optimum composition based on trial experiments, where agglomeration could be avoided. Furthermore, a previous study has also reported that higher weight fractions may cause more agglomeration of silver nanoparticles, which affected larger fiber diameters and rough surfaces [26,33].

Figure 2a,b show swelling effects among the fibers which resulted into non-uniform sizes of diameters, connected to every fiber. This was caused by several factors in fiber fabrication via electrospinning such as polymer concentration, viscosity, voltage, and polymer rate, as well as rotational speed and distance from the collector. One concern was that the collector distance being too close would result in presence of solvent between the fibers since the solvent could not evaporate completely when deposited in the collector. This causes the fibers to overlap parallel to each other and to partially form junctions [25]. Increasing the collector distance (in this present work, 10 cm of distance has successfully produced the nanofibers) would increase the time for the solvent to evaporate. Moreover, the elongation of the polymer fibers could occur more optimally to produce finer and more uniform fibers [34]. Therefore, the formation of electrospun nanofibers could establish layers with evenly distribution of silver within this 10 cm-distance for the next calcination step.

In contrast, the diameters of PVA and PVA/AgNO_3_ iodinated fibers had twice the diameter of non-iodinated fibers as shown in Figure 2c,d. The average diameter of iodinated PVA was 189.66 nm, whereas for the iodinated PVA/AgNO_3_ the average diameter was 204.21 nm. The increase is due to the swelling effect caused by iodine absorption into PVA fibers [22]. As a result, the conversion of polyol with hydrophilic properties to polyene with hydrophobic properties took place [14,18,23]. 

The effect of AgNO_3_ addition into PVA was the presence of silver nanoparticle in PVA as the reductor with the presence of -OH [14]. Consequently, in PVA/AgNO_3_ fibers, the swelling aggregates as shown in Figure 2d, indicated the formation of AgI aggregates during iodination process. This was in accordance with the results reported by Sheha et al. (2012) who utilized copper and PVA as the source for electrolyte polymer which later was iodinated [35]. As long as swelling fibers in PVA and PVA/AgNO_3_ were coincided, cross-linking reactions could have happened during the iodination process [36].

**Figure 3:**
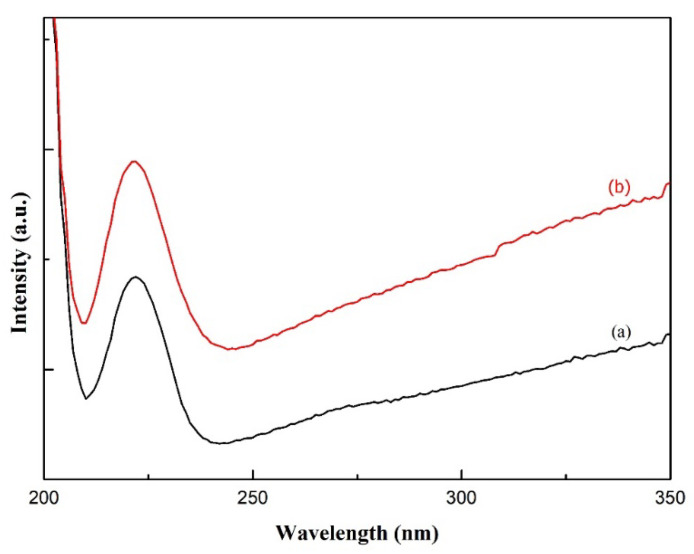
UV-Vis Spectra of carbon nanofibers from (**a**) PVA and (**b**) PVA/AgNO_3_.

A noticeable difference between PVA and PVA/AgNO_3_ carbon fibers could be observed in Figure 1d,e. Sharp and narrow peaks observed at 1627 cm^−1^ in the carbon fibers, indicating the presence of aromatic cyclic –C=C–, so sp^2^ hexagonal carbon structure was confirmed [18]. In addition, the determination of π-π* adsorption band in C=C aromatic cyclic was conducted via UV-Vis Spectrophotometry (Figure 3). As can be seen, the transition occurred at about 225 nm, which is in agreement with [36], who reported a range between 230 and 270 nm and Woodward-Fieser rules who suggested a value equal to 245 nm [37]. Peaks to indicate Ag^0^ in the interval of 400–420 nm were not observed, as the amount of AgNO_3_ samples was small. Thus, the confirmation of Ag^0^ existence was carried out by using TEM.

**Figure 4:**
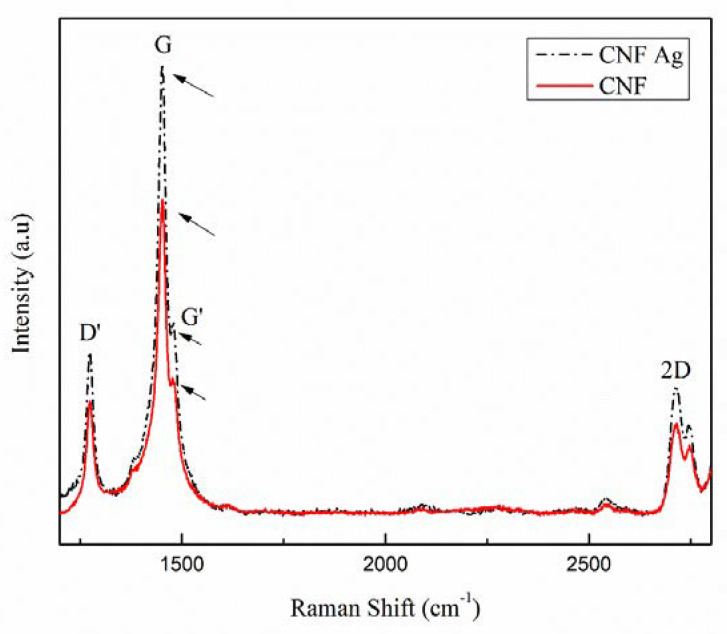
Raman shifts of carbon nanofibers (CNF) with and without the addition of silver nanoparticle (SNP).

The determination of graphitic structure in carbon fibers was performed via Raman spectrophotometry presented in Figure 4. Carbon nanofiber (CNF) with Ag confirmed the presence of first and second order of D bands at the peak in 1400–1500 cm^−1^ interval, whereas the first order of G-band that showed π-π* of sp^2^ carbon structures were observed at higher intensities. As a comparison between the second order of G-band in Raman spectra with the word 2D as the indicator, the peak of G^I^ was in lower intensities than that in G-band. An increase in the G-band peak intensities of CNF Ag implied to an increase in carbon fibers yield as well as the width, which shows high crystalline structure of carbon structures. These Raman spectra results have confirmed that the introduction of silver to the system could lead to the formation of graphene layers with high yield. A study conducted by Fatemah et al. (2011) has suggested the graphene layers are formed due to the introduction of Ni as the metal catalyst; however, their Raman spectra shows relatively low crystalline structure (wider shifting) [14].

**Figure 5:**
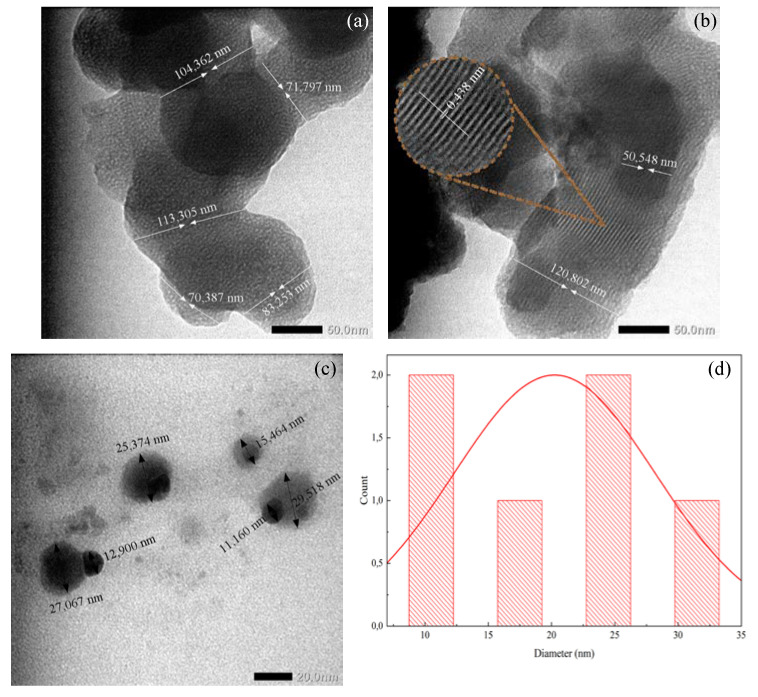
TEM images of (**a**) Carbon nanofiber from iodinated PVA nanofiber, (**b**) carbon nanofiber with silver nanoparticle from iodinated PVA/AgNO_3_, (**c**) silver nanoparticle, and (**d**) diameter particle distribution.

Iodinated PVA and PVA/AgNO_3_ nanofibers were prepared by pyrolysis. The silver nanoparticles were in spherical shapes with sizes around 10–35 nm (Figure 5). The calcination that occurred at 500 °C (at most) for 6 h has successfully altered the fibrous structure into layers of graphene fibers with an average diameter of 88.62 nm to 120 nm. Figure 5a,b display TEM images of PVA and PVA/AgNO_3_ carbon fibers, respectively, and the insert in Figure 5b depicts the layers of graphene that were successfully synthesized from the samples.

The one-step heating treatment in this study was able to reduce the diameter of iodinated electro-spun fibers by half compared to those of the non-iodinated samples without significantly changing the fiber structures. As shown in SEM and TEM images in Figure 2 and Figure 5, respectively, the diameters of fibers reduced from 189.66 nm to 100 nm and 204.21 nm to 120 nm for PVA and PVA/AgNO_3_ samples, respectively. The decrease in diameters took place during pyrolysis by removing volatile components and dehydrating the fibers, followed by the rearrangement of graphene layers to form more concise structures [22]. These findings also highlight that the presence of silver nanoparticles resulted in more regular graphene layers than samples without silver nanoparticles. This observation is in agreement with a previous study, which reported that the use of metals formed more regular graphene layers [14]. The analysis of ImageJ software to measure the distance between the formed layers was around 0.438 nm on Figure 5d.

Structural changes in PVA, such as (–OH) into polyene (–C=C–) appeared with the iodination process due to dehydration and elimination reactions [14,22,23,38]. As a result, the hydrophilic characteristics were replaced by the hydrophobic properties, where the cyclization and aromatization in the polycyclic carbon yields were higher than the -OH group and eventually provided more stable thermal properties [14,18,23,39]. Furthermore, this mechanism occurred at higher temperature and tended to form aromatic rings easily through Diels–Alder reaction, where small bands in PVA/AgNO_3_ were reduced as shown in FTIR spectra in Figure 1b,c. This reduction occurs because the some OH and C–O– from PVA bound to Ag from AgNO_3_ [39]. Although the electro-spun fibers and silver nanoparticle addition resulted in the increase in yield, further investigation of various AgNO_3_ compositions in electro-spun step needs to be carried out to determine the optimum silver nanoparticle content for maximizing the carbon nanofiber yield.

**Figure 6:**
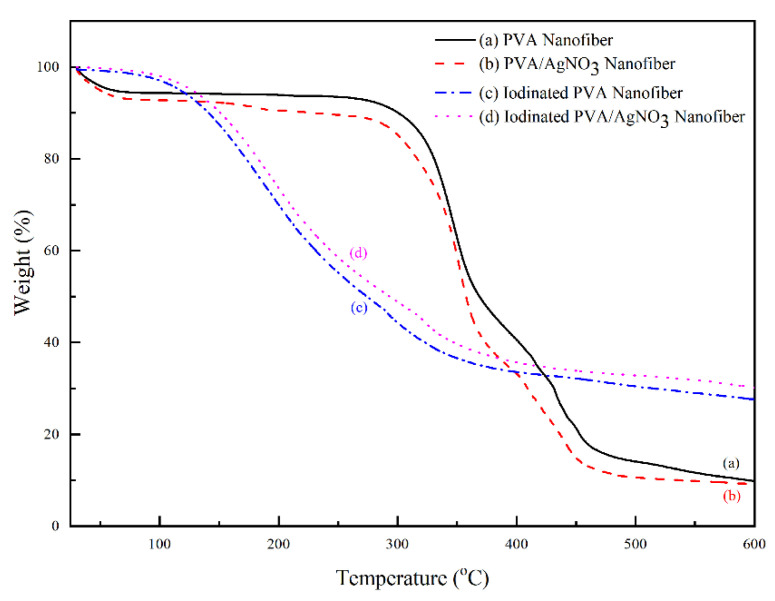
Thermal gravimetric pattern of PVA and PVA/AgNO_3_ samples based on iodination treatment.

**Figure 7:**
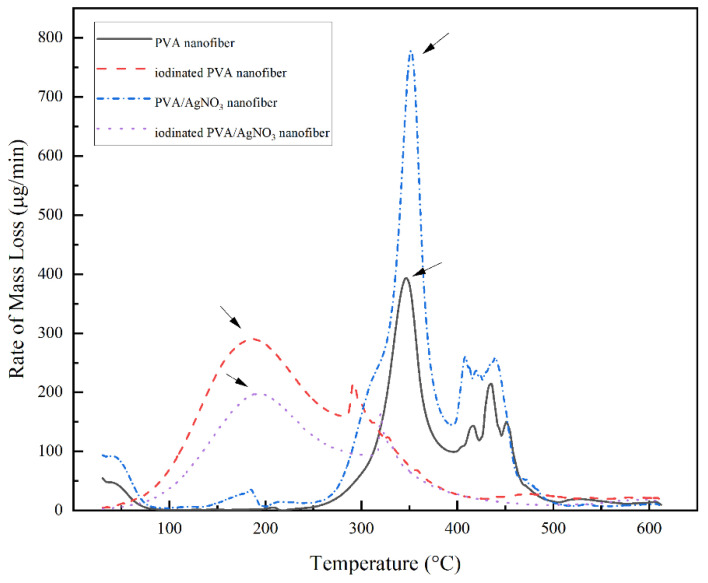
DTG thermogram of PVA Nanofiber, Iodinated PVA Nanofiber, PVA/AgNO_3_ Nanofiber, and Iodinated PVA/AgNO_3_ Nanofiber.

The change of mass with increasing temperature can be seen in Figure 6. The iodinated samples had similar mass loss behavior. The decomposition began above 100 °C and completed at 350 °C with 40% residual mass. The decomposition of PVA continued up to 600 °C with only 33% remaining as the final residue. In the below results (Figure 7), the PVA/AgNO_3_ nanofiber showed the maximum rate of mass loss at 350 °C, which is almost doubled compared to the cases where AgNO_3_ is absent, and this shows the effect of SNP within the electrospun of PVA/AgNO_3_. Without the presence of SNP, extensive degradation of PVA occurred above 400 °C [40]; however, the presence of SNP shifted the initial degradation temperature below 300 °C. Based on the DTG, the maximum temperature of degradation of the polymeric backbone occurred at 350 °C, which indicated a significant decrease of the degradation temperature. Compared to a study that utilized Ni metals, the degradation occurred above 500 °C [14,41]. Whilst, in iodinated PVA/AgNO_3_ sample, the presence of Ag^0^ in PVA increased the barrier energy. Therefore, the degradation of polymeric chains, as well as the formation of olefin, were delayed and resulted in lower mass loss [39]. In contrast, the non-iodinated samples had only 10% of residue left from the total mass (Figure 6). This significant loss shows that PVA was thermally unstable and could be decomposed into lower molecular constituents with volatile characteristics [14].

## 4. Conclusions

The synthesis of carbon nanofibers from PVA, with the addition of silver nanoparticles in the form of AgNO_3_ via one-step pyrolysis treatment, was successfully performed. Two important features in this research were the implementation of iodination electrospinning and AgNO_3_ to produce highly thermally stable properties at 350 °C on 800 µg/min, which are required to form graphene layers (in average for 0.348 nm), as well as to increase yield based on Raman spectroscopy results. Our study has confirmed that these two pre-treatments had improved the yield by three times compared to the samples without AgNO_3_. Based on the TGA results, the presence of graphene layers was obtained at significantly lower temperature, which was at 400 °C with around 33% of residual. Moreover, samples without electrospinning and iodination procedures showed lower amount of graphene layers with 10% of residue.

## Figures and Tables

**Table 1:** Conductivity and viscosity of PVA and PVA/AgNO_3_ solutions.

No.	Polymer	Density (Kg/m^3^)	Viscosity		Conductivity (µs/cm)
			(Nm/s^2^)	Cp	
1	PVA 13 w/v%	1.106	0.005711	5.711	166.5
2	PVA 13 w/v%/AgNO_3_ 0.2 w/t%	1.128	0.008072	8.072	472.6

## Data Availability

All data generated and analysed during this study are included in this published article (and its Supplementary Information Files).

## Supplementary Materials

The following are available online at https://www.mdpi.com/article/10.3390/polym14030446/s1, Figure S1: Ionic interaction within PVA polymer solution, Figure S2: SEM photographic images, Figure S3: Possible model structure on the formation of graphene, Table S1: The density, viscosity, and conductivity measurement.
