# Supplementary material for: Carbon-Nano Fibers Yield Improvement with Iodinated Electrospun PVA/Silver Nanoparticle as Precursor via One-Step Synthesis at Low Temperature
Source: Polymers (Basel). 2022 Jan 22;14(3):446. doi: 10.3390/polym14030446 (PMC8839439; doi:10.3390/polym14030446)
Supplement: Supplementary file 1 [file polymers-14-00446-s001.zip › polymers-1506971-supplementary.pdf]

Supporting Information

Carbon-Nano Fibers Yield Improvement with Iodinated Electrospun PVA/Silver Nanoparticle as Precursor Via One-Step Synthesis at Low Temperature

Saharman Gea[1,2*], Boy Attaurrazaq[1,2], Suhut A Situmorang[1,2], Averroes F Piliang[1,3], Sunit Hendrana[4], Stergios Goutianos[5]

[1]Cellulosic and Functional Materials Research Centre, Universitas Sumatera Utara, Jl. Bioteknologi No. 1, Medan 20155, Indonesia

[2]Department of Chemistry, Faculty of Mathematics and Natural Sciences, Universitas Sumatera Utara, Jl. Bioteknologi No. 1, Medan 20155, Indonesia

[3]Department of Physics, Faculty of Mathematics and Natural Sciences, Universitas Sumatera Utara, Jl. Bioteknologi No. 1, [1]Medan 20155, Indonesia

[4]Research Centre for Chemistry, Indonesian Institute of Sciences (LIPI), Kawasan Puspitek Gedung 452, Serpong, Tangerang Selatan, Banten, 15314, Indonesia

[5]Department of Manufacturing and Civil Engineering, Norwegian University of Science and Technology, Gjovik, Norway

Table S1. The density, viscosity, and conductivity measurement of 13% of PVA and 0.2 w/t% of PVA/$AgNO_3$.

No	Polymer	Density (Kg/m^3)	Viscosity		Conductivity (µs/cm)
			(Nm/s^2)	cP	
1	PVA 13 w/v%	1,106	0,005711	5,711	166,5
2	PVA 13 w/v%/$AgNO_3$ 0,2 w/t%	1,128	0,008072	8,072	472,6

As a higher concentration of polymer affects the level of viscosity itself, the addition of silver (Ag^+) and nitrate (NO_3^-) from the salt could promote the process of van der waals interaction between hydroxyl groups from PVA which are partially negative and silver ions which are partially positive. On the other hand, the interaction of hydrogen bonds between hydroxyl from PVA and nitrate anions (NO_3^-) could also occur. Thus, these interactions could cause the surface tension on the polymer to be higher due to the increase viscosity (Abdel-

*Corresponding author. Cellulosic and Functional Materials Research Centre (CFM-RC), Universitas Sumatera Utara, Jl. Bioteknologi No. 1, Medan 20155, Indonesia.

***e-mail address*: s.gea@usu.ac.id (Saharman Gea)**

Mohsen et al., 2019). The presence of silver nitrate ($AgNO_3$) adds higher density of ions/ charges (Ag^+ and (NO_3^-))in the polymer, so that it also improves in addition the electrical properties of the polymer as it is shown in the following Figure S1.

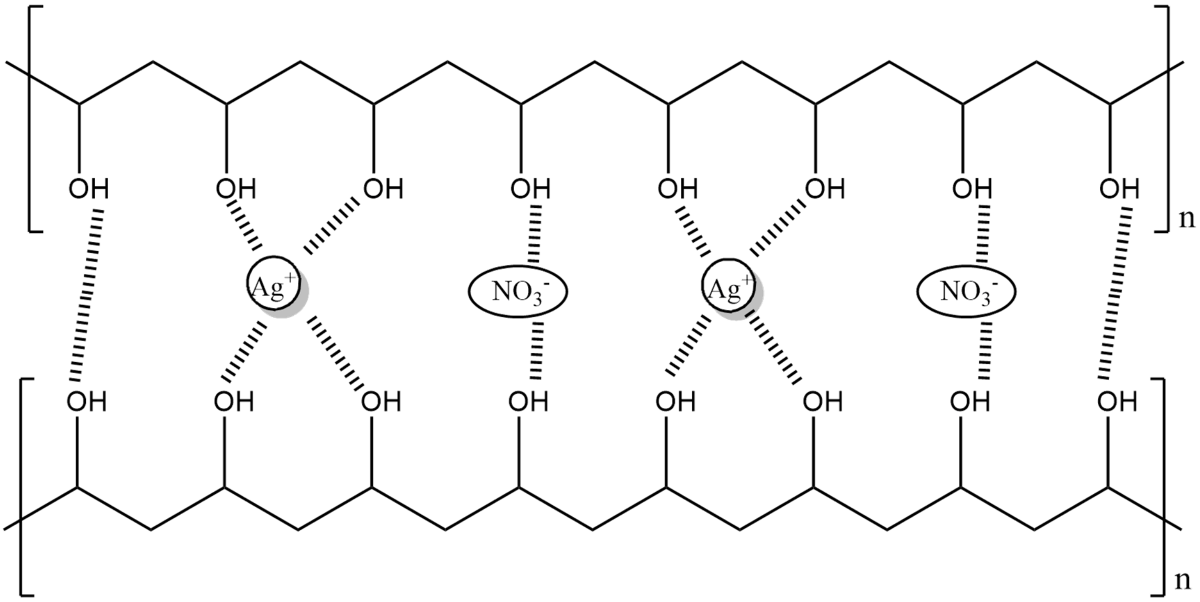

Figure S1. Ionic interaction in the PVA polymer solution (Abdel-Mohsen et al., 2019).

The decrease of fiber diameter during the pyrolysis occurred due to the dehydration steps and the removal of volatile chemicals as well as the reduction of graphene layers into smaller structures (Gupta dan Dhakate, 2017). With the presence of SNP, the graphene structure seems to be more uniform and longer compared to the PVA without the SNP. The Figure S2 shows the formation of graphene formation.

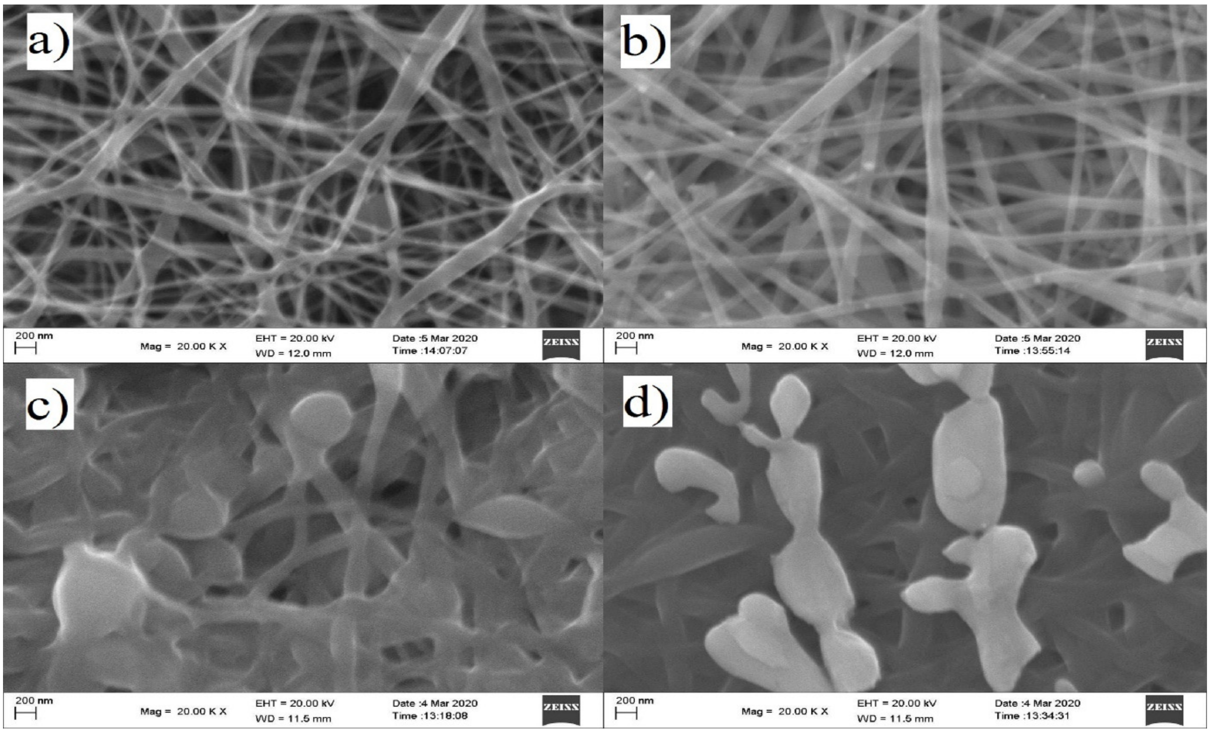

Figure S2. SEM Photographic Images a) PVA Nanofiber; b) PVA/$AgNO_3$ nanofiber; c) Iodinated PVA Nanofiber, d) Iodinated PVA/$AgNO_3$ Nanofiber of 20.000 enlargement.

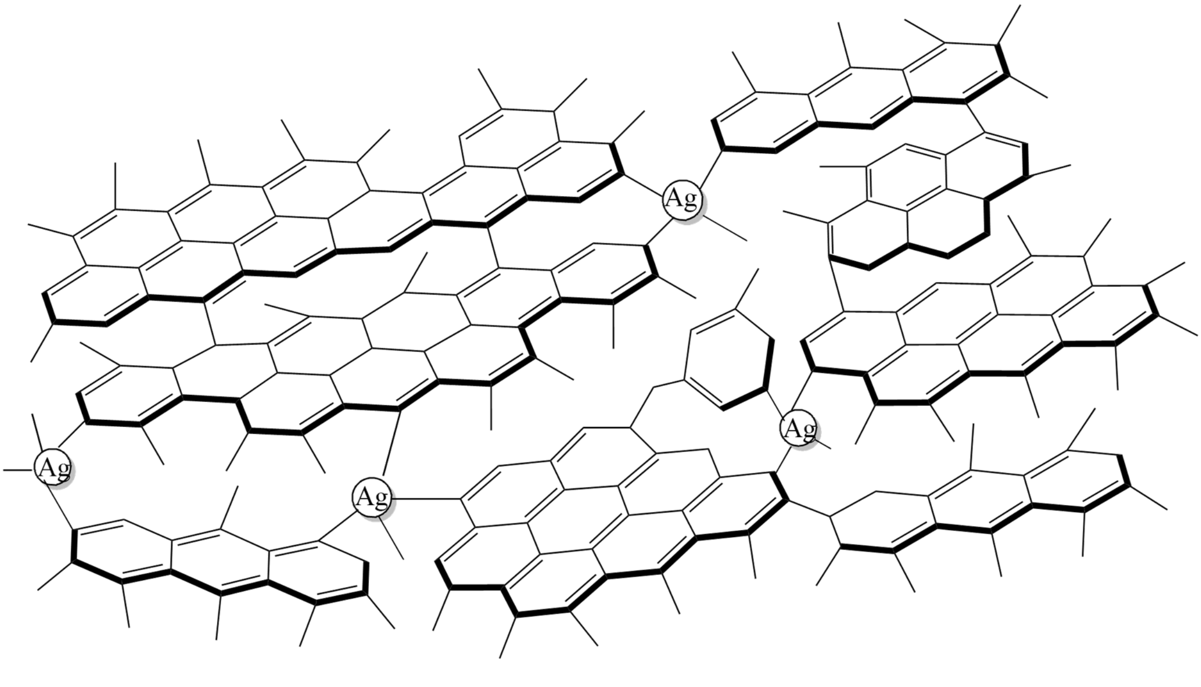

Figure S3. Possible model structure on the formation of graphene layers with the presence of silver nanoparticle (Fatema et al., 2011b).
